# A Framework for Regularized Non-Negative Matrix Factorization, with Application to the Analysis of Gene Expression Data

**DOI:** 10.1371/journal.pone.0046331

**Published:** 2012-11-02

**Authors:** Leo Taslaman, Björn Nilsson

**Affiliations:** 1 Department of Hematology and Transfusion Medicine, Lund University, Lund, Sweden; 2 Broad Institute, Cambridge, Massachusetts, United States of America; University of Manchester, United Kingdom

## Abstract

Non-negative matrix factorization (NMF) condenses high-dimensional data into lower-dimensional models subject to the requirement that data can only be added, never subtracted. However, the NMF problem does not have a unique solution, creating a need for additional constraints (regularization constraints) to promote informative solutions. Regularized NMF problems are more complicated than conventional NMF problems, creating a need for computational methods that incorporate the extra constraints in a reliable way. We developed novel methods for regularized NMF based on block-coordinate descent with proximal point modification and a fast optimization procedure over the alpha simplex. Our framework has important advantages in that it (a) accommodates for a wide range of regularization terms, including sparsity-inducing terms like the 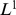 penalty, (b) guarantees that the solutions satisfy necessary conditions for optimality, ensuring that the results have well-defined numerical meaning, (c) allows the scale of the solution to be controlled exactly, and (d) is computationally efficient. We illustrate the use of our approach on in the context of gene expression microarray data analysis. The improvements described remedy key limitations of previous proposals, strengthen the theoretical basis of regularized NMF, and facilitate the use of regularized NMF in applications.

## Introduction

Given a data matrix 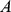 of size 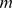
×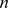, the aim of NMF is to find a factorization 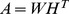 where 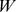 is a non-negative matrix of size 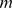
×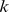 (the component matrix), 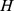 is a non-negative matrix of size 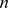
×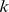 (the mixing matrix), and 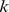 is the number of components in the model. Because exact factorizations do not always exist, common practice is to compute an approximate factorization by minimizing a relevant loss function, typically
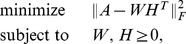
(1)where 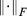 is the Frobenius norm. Other loss functions include Kullback-Leibler’s, Bregman’s, and Csiszar’s divergences [1]–[4]. Problem 1 has been well studied and several solution methods proposed, including methods based on alternating non-negative least squares [5], [6], multiplicative updates [1], [3], [7], [8], projected gradient descent [9]–[11], and rank-one residue minimization [12] (reviews in refs. [9], [13]).

The NMF problem is computationally hard. Particularly, an important property is that the factorization is not unique, as every invertible matrix 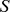 satisfying 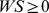 and 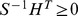 will yield another non-negative factorization 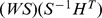 of the same matrix as 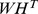 (simple examples of 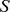 matrices include diagonal re-scaling matrices) [14]. To reduce the problem of non-uniqueness, additional constraints can be included to find solutions that are likely to be informative/relevant with respect to problem-specific prior knowledge. While prior knowledge can be expressed in different ways, the extra constraints often take the form of regularization constraints (regularization terms) that promote qualities like sparseness, smoothness, or specific relationships between components [13]. At the same time, the computational problem becomes more complicated, creating a need for computation methods that are capable of handling the regularization constraints in a robust and reliable way.

We developed a novel framework for regularized NMF. This framework represents an advancement in several respects: first, our starting point is a general formulation of the regularized NMF problem where the choice of regularization term is open. Our approach is therefore not restricted to a single type of regularization, but accommodates for a wide range of regularization terms, including popular penalties like the 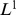 norm; second, we use an optimization scheme based on block-coordinate descent with proximal point modification. This scheme guarantees that the solution will always satisfy necessary conditions for optimality, ensuring that the results will have a well-defined numerical meaning; third, we developed a computationally efficient procedure to optimize the mixing matrix subject to the constraint that the scale of the solution can be controlled exactly, enabling standard, scale-dependent regularization terms to be used safely. We evaluate our approach on high-dimensional data from gene expression profiling studies, and demonstrate that it is numerically stable, computationally efficient, and identifies biologically relevant features. Together, the improvements described here remedy important limitations of earlier proposals, strengthen the theoretical basis of regularized NMF and facilitate its use in applications.

## Results

### Regularized Non-negative Matrix Factorization with Guaranteed Convergence and Exact Scale Control

We consider the regularized NMF problem
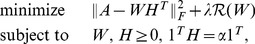
(2)where 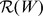 is a regularization term, 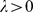 determines the impact of the regularization term, and 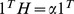 is an extra equality constraint that enforces additivity to a constant 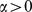 in the columns 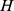. While we have chosen to regularize 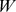 and scale 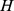, it is clear that the roles of the two factors can be interchanged by transposition. We assume that 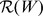 is convex and continuously differentiable, but do not make any additional assumptions about 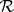 at this stage. Thus, we consider a general formulation of regularized NMF where one factor is regularized, the scale of the solution is controlled exactly, and the choice of regularization term still open.

The equality constraint that locks the scale of 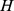 is critical. The reason is that common regularization terms are scale-dependent. For example, this is the case for 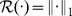 (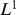/LASSO regularization), 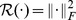 (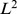/Tikhonov regularization), and 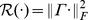 (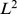 regularization with an inner operator 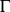 that encodes spatial or temporal relationships between variables). Scale-dependent regularization terms will pull 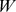 towards zero, and indirectly inflate the scale of 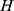 unboundedly. Locking the scale of the unregularized factor prevents this phenomenon.

To solve Problem 2, we explored an approach based on block coordinate descent (BCD). In general, the BCD method is useful for minimizing a function 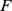 when the coordinates can be partitioned into 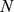 blocks such that, at each iteration, 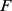 can be minimized (at low computational cost) with respect to the coordinates of one block while the coordinates in the other blocks are held fixed. The method can be expressed as the update rule
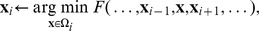
where 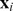 and 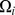 denote the coordinates and domain of the 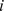
th block, respectively. The updates are applied to all coordinate blocks in cyclic order. In the case of NMF, there are three natural ways to define blocks: per-column, per-row, or per-matrix. We partition the coordinates of 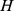 per column whereas the partitioning of 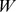 depends on the anatomies of 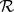 and the subproblem solver (details below).

Regarding the convergence of BCD procedures, it can be shown that if the domain for the 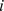
th coordinate block, 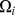, is compact and all subproblems are strictly convex (that is, 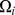 is convex and 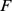 is strictly convex over 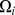), the sequence generated by a BCD procedure has at least one limit point and each limit point is a critical point of the original function 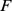 [15]. In this context, we say that an algorithm has converged if the current point is within a tolerance from a critical point (that is, a point 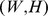 where the derivative of the objective function is non-negative in all feasible directions; the first-order necessary condition for optimality). If 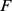 is convex but no longer strictly convex in 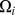, limit points are still guaranteed to exist but are not necessarily critical points (that is, the solution may not satisfy the first-order criterion for optimality).

In Problem 2, the clamping of the scale bounds 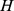 and, indirectly, also 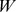. Hence, all 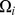
’s are bounded. Because they are also closed, they are compact. However, subproblems that are not strictly convex may still occur. To guarantee solutions that represent critical points, we therefore need to safeguard against non-strict convexity in the BCD subproblems. To this end, we add a *proximal point term* to objective functions of subproblems that are not known to be strictly convex beforehand. A proximal point term penalizes the Euclidean distance to the previous point in 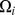, makes the subproblems strictly convex, and guarantees that limit points of the generated sequence are critical points of the *original* function 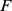 [16]. The BCD updates change to
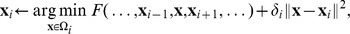
where 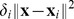 is the proximal point term and 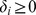 a small number which can be zero if 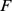 is known to be strictly convex in 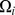 (in this case the proximal point term is not needed).

### Optimizing the Mixing Coefficients

We developed an efficient procedure to optimize each block (column) of the mixing matrix 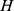. The procedure itself is given in Algorithm 1. This section describes the proof.

The constraints 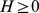 and 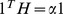 imply that columns of 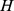 must lie in the 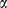 *-simplex*, defined as
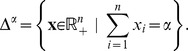



Geometrically, this is the intersection of the non-negative orthant and a hyperplane with normal vector 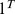 and offset 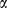 from the origin. The set is convex and also compact, meaning the conditions for a BCD to converge discussed in the previous section are satisfied.

We first derive general optimality criteria for convex functions on 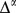. Let 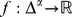 be convex and differentiable. By definition, 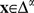 is a minimum of 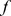, if and only if the directional derivative at 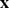 is non-negative in every feasible direction
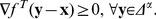
(3)


Considering the special cases 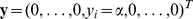, we see that
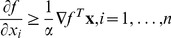
(4)must hold if 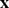 is a minimum. However, the converse also holds. Assuming that Equation 4 holds and letting 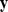 be an arbitrary point in 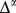, we have



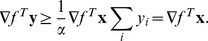



Hence, Equation 3 and Equation 4 are equivalent. Moreover, Equation 4 can be rephrased as
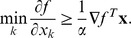



This is interesting because the fact that 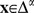 implies that the reversed inequality also holds
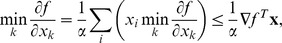
meaning we have inequality in both directions, meaning 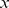 is a minimum if and only if



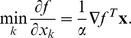



The right-hand side of this equation is a weighted average of partial derivatives. Because the weights are non-negative and the smallest partial derivative is included when forming this average, all partial derivatives that correspond to non-zero coordinates of 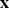 must equal the smallest partial derivative at 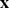. Taken together, 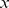 is a minimum of a convex function 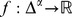 if and only if
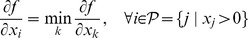
(5)where 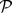 denotes the indices of the non-zero coordinates in 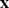. This somewhat surprising result sets the stage for the development of an efficient way to minimize the columns of 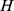.

We next connect Equations 2 and 5 using a rank-one residue approach. Rewriting 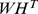, we have
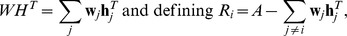
the subproblem of updating a column 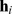 becomes



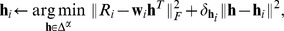
which is the same as
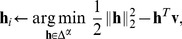
(6)where 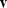 denotes the constant vector 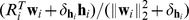. The key to solving this problem efficiently lies in the observation that 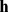 can be solved directly when the indices of the non-zero variables are known. To see this, assume for a while that 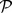 is given and let 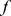 be the above objective function of Problem 6. Because 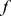 is convex, Equation 5 implies that all its partial derivatives with respect to the non-zero variables share a common value, that is



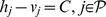
for some 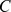 at the minimum. Summing over 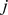 and using the fact that 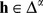, we can solve for 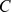




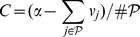
meaning 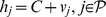, 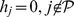. Thus, all that remains is a way to find 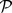. Although this may seem like a problem with a complexity of 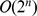 at first sight, it turns out that 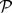 must correspond to the indices of the 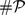 largest coordinates of 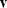. To see this, assume that 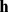 is a minimum and that there exist indices 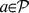 and 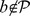 such that 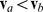. Then, the entries 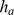 and 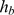 could be swapped to obtain another feasible vector that would yield a smaller objective function value in Equation 6, contradicting that 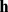 is a minimum. Hence, the only remaining question is how many coordinates are non-zero at the minimum. This question can be resolved by computing 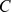 and the partial derivatives for different values of 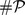 until Equation 5 is satisfied. This procedure can be implemented as a linear 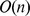 search (Algorithm 1) and is amenable to speed-ups when used iteratively (Discussion).

### Optimizing the Components

Unlike the optimization of 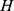, which is independent of 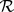, the optimization of 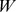 depends on the choice of 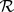. We next give 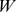 optimization procedures for three common types of regularization:

#### Sparseness regularization

A common way to enforce sparsity is to penalize the 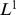 norm, the closest convex 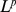 relaxation of the 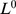 penalty (the number of non-zero elements). To optimize 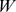 with 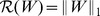, one possibility is to use the rank-one residue approach. Rewriting 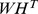 as a sum of rank-one matrices and considering the Karush-Kuhn-Tucker (KKT) conditions, it is easy to show that the BCD update for the column/block 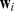 is given by
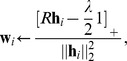
where 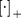 denotes truncation of vector elements at zero. Another possibility is to view 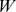 as a single block, in which case the minimization can be rewritten as a non-negative least squares problem (this follows directly from the KKT conditions) that can be solved efficiently using for example the Fast Non-Negative Least Squares algorithm (FNNLS) [17].

#### Tikhonov regularization

We next consider 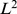 regularization with 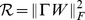 where 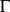 is an 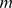
×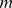 filter matrix. This type of regularization is used to impose various types of smoothing, for example by using 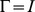 or various difference operators, like 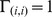, 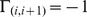, and 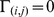 elsewhere. Partitioning the coordinates per column and using a rank-one residue approach, the column-wise BCD updates become
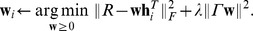



Expanding the norm and removing constant terms, we get
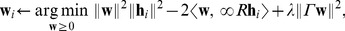
(7)which is a non-negative least squares problem. To see this, let 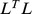 be the Cholesky decomposition of 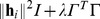 and consider



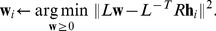
(8)Expanding Equation 8 and removing the constant term, we recover Equation 7. Hence, we can solve Equation 8 which can be done using non-negative least squares algorithms that start from the normal equations and do not require explicit Cholesky decomposition [17]–[19].

#### Related base vector regularization

In some applications, certain base vectors are known to be closer to each other. For example, this type of regularization may be motivated in the reconstruction of cell type-specific gene expression profiles from gene expression profiles of compound tissues, where the gene expression patterns of related cell types can be expected to be similar. One way to incorporate such information is to penalize the squared distance between base vectors that are known to be related. The objective function becomes
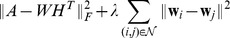
where the set 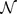 defines pairs of adjacent vectors, encoded as a matrix 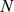 where each column defines a pair 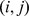 by having elements that are 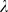 at position 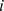 and 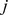 and 0 elsewhere. The objective function can then be written as



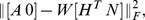
the minimum of which with respect to 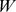 can again be found using FNNLS or other non-negative least squares algorithms.

### Computational Efficiency

To illustrate its use, we implemented our method with 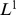 norm-induced sparseness regularization (Algorithm 2; denoted rNMF), and applied it to sets of gene expression profiles of blood disorders (Table 1). For comparison, we considered two previously published methods [20], [21]. These methods are relevant as control methods as they also seek to perform NMF with 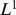 regularization and exact scale control. Other sparse NMF methods have been published (Discussion), but solve different formulations and, hence, are less relevant as controls in this context. Out of the two selected control methods, we found the method in [21] to be the most efficient, making it a representative control method. Each data set was analyzed with different numbers of components (k = 5,10, and 15) and regularization parameter values (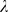 selected to yield 25%, 50%, and 75% zeroes in 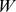; the value needed to achieve a specific degree of sparsity varies between data sets).

**Table 1:** Time (seconds) needed to complete one update of all coordinates and to reach convergence in sets of gene expression data from blood disorders.

		rNMF		control	
Data set, reference	Data size	iteration	convergence	iteration	convergence
Acute Myeloid Leukemia [36]	22283×293	0.75	21.7	2.2	219.4
Acute Myeloid Leukemia [37]	54613×461	3.95	128.8	10.2	>600
Acute Myeloid Leukemia [38]	44692×162	0.96	17.3	1.5	163.6
Acute Lymphoblastic Leukemia [39]	22215×288	0.94	17.8	2.3	245.7
Multiple Myeloma [40]	54613×320	3.04	29.1	6.4	>600

All methods were implemented in C++ and identically initialized. Timings obtained on a 2.30 GHz Intel Core i7 2820QM CPU with 16 GB RAM. For convergence, we required a relative decrease in the objective function less than 10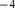 in successive iterations. Throughout, 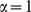 and 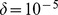.

**Figure 1:**
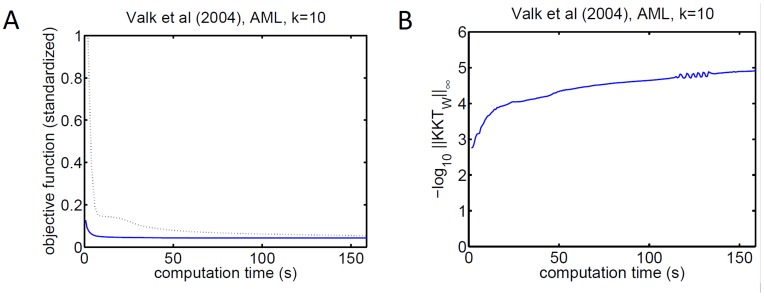
Convergence of rNMF on real data. Left: The objective function decreases faster with rNMF (blue) than the control method (dashed). We standardized the objective function by dividing it by the squared Frobenius norm of 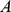. Right: As predicted theoretically, rNMF closes the KKT conditions (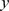 axis indicates the negative logarithm of the max-norm of the KKT condition matrix for 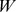, that is 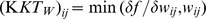 which should approach the zero matrix). The results in this figure were obtained for gene expression profiles of Acute Myeloid Leukemia [36], 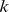 = 10, and 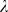 set to yield about 50% sparsity. This example is representative as similar results were obtained for other data sets and parameter choices.

Throughout, rNMF was 1.5 to 3.0 times faster per iteration and converged considerably faster (Table 1 and Figure 1a). The method also exhibited robust closing of the KKT conditions, illustrating that the theoretical prediction that solutions represent critical points holds numerically in practice (Figure 1b).

### Analysis of Gene Expression Data

To illustrate the use of our approach in a practical situation, we applied rNMF to the Microarray Innovations in LEukemia (MILE) data set [22], [23], containing 2096 gene expression profiles of bone marrow samples from patients with a range of blood disorders (Affymetrix Human U133 Plus 2.0 arrays; 54612 genes expression values/probes per sample). We applied rNMF to the MILE data with varying numbers of components (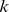 = 10, 20 and 30) and varying degrees of sparsity (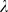 chosen to yield 50%, 75%, and 90% sparsity in 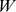). To illustrate the effect of sparsity regularization, we also analyzed the data using conventional NMF (equivalent to setting 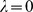).

Now, it is well known that the bone marrow morphology varies considerably between disorders and between patients, especially in terms of the abundances of various classes of blood cells. It is also known that different classes of blood cells exhibit distinct gene expression patterns [24]. Much of the variation in the data will therefore be caused by fluctuations in cell type abundances and by differences in gene expression between cell types. Because rNMF and NMF are driven by variation, it is reasonable to assess the biological relevance of the results by testing whether the components contain gene expression features belonging to specific classes of blood cells. To this end, we used gene set enrichment testing, a statistical technique that is widely used in genomics to annotate high-dimensional patterns. In essence, statistically significant enrichment for a gene/probe set in a component means that the genes/probes comprising the set have higher coordinate values (at the set level) in a component than would be expected by chance (*c.f.*, ref. [25], [26]). We defined sets of marker genes for all major classes of blood cells (Materials and Methods), and tested for enrichment of each of these sets in each component using the program RenderCat [25].

**Figure 2:**
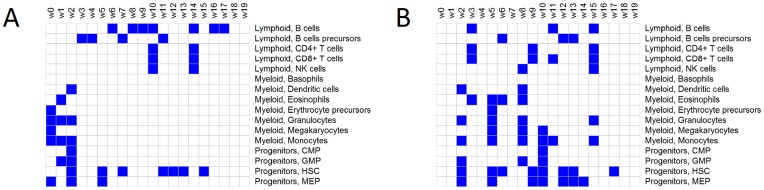
Application to gene expression microarray data from blood disorders. Columns indicate components, rows classes of blood cells. Blue cells indicate significant enrichment of cell type-specific markers (as detected by gene set enrichment testing; 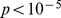) in the component generated by rNMF with 90% sparsity (**a**) and conventional NMF (**b**). The components have been ordered by strength (defined as 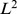 norm of 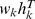) with 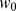 denoting the strongest component. As discussed in detail in Results, strong components generated by rNMF capture cell type-related gene expression features more clearly than conventional NMF.

As illustrated in Figure 2a, enrichments of cell type markers were identified in all rNMF components except the weakest ones. Enrichments of markers for almost all major cell types in the bone marrow were detected in at least one component. In some components, enrichments of markers belonging to multiple cell types were detected. In these cases, the detected cell types belonged to the same developmental lineages (and hence have similar gene expression patterns). For example, this can be seen in Figure 2a where 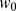, 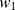, and 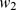 are enriched for features from multiple myeloid cell types and 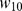 and 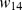 enriched for features from multiple lymphoid cell types. Together, the results support that the components are biologically relevant.

Conventional NMF also generated components with enrichments of cell type-specific markers. Interestingly, however, we observed differences as to which components did and did not capture cell type-specific features. As shown in Figure 2a, strong components generated by rNMF could usually be annotated and components that could not be annotated were usually the weakest ones. With conventional NMF, this pattern was generally not seen. Instead, as shown in Figure 2b, strong components could often not be annotated, suggesting that conventional NMF did not enrich for cell type-specific features. A likely explanation could be that there are relatively few cell type-specific markers compared to the number of genes in the genome, and that limiting the cardinality of components by including 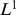 regularization promotes the identification of small sets instead of broader features that are less specific.

## Discussion

Non-negative matrix factorization has been previously suggested as a valuable tool for analysis of various types of genomic data, particularly gene expression data [27]–[31]. The rationale is that gene expression is an inherently non-negative quantity. In this case, NMF allows the data to be expressed in their natural scale, thereby avoiding re-normalization by row-centering as is needed by dimension-reduction techniques based on correlation matrices (*e.g.*, principal component analysis).

We developed methods that enable robust and efficient solution of a range of regularized NMF problems and tested these methods in the context of gene expression data analysis. The key component of our approach is an efficient procedure to optimize the mixing coefficients 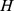 over the 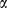-simplex, enabling the scale of the solution to be explicitly controlled. Further, our approach separates the task of optimizing 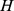 and optimizing 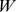. This has three advantages. First, the optimization of 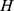 becomes independent of the regularization term, meaning the same algorithm (Algorithm 1) can always be used. Second, as exemplified by the 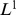 regularization case, the optimization of 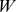 is simplified, at least with standard regularization terms. Third, a proximal point term can be included, guaranteeing convergence towards critical points, ensuring that the results will always have well-defined numerical meaning [16]. Experimentally, we have illustrated that our method is computationally efficient and capable of enhancing the identification of biologically relevant features from gene expression data by incorporating prior knowledge.

Previous work on regularized NMF is limited compared with previous work on conventional NMF. A straightforward formulation is
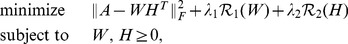
(9)where are the functions 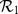 and 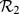 enforce the regularization constraints, and the parameters 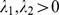 control the impact of the regularization terms [13]. This formulation allows regularization of both factors and basic computation methods can be derived for some choices of 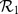 and 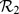 by extending conventional NMF methods [32], [33]. However, balancing 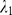 and 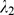 against each other is often difficult and simultaneous regularization of both factors is rarely wanted. More commonly, the goal is to regularize one of the factors. For example, to get sparse component vectors, an 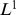 penalty can be imposed on 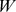 whereas 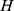 does not have to be regularized. In Equation 9, single-factor regularization would correspond to setting 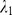 or 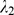 to zero. Again, with standard scale-dependent regularization terms, this will pull the regularized factor towards zero and inflate the unregularized factor unboundedly. Scale-independent penalty terms have been proposed [34], but these are non-convex and therefore complicate optimization with respect to the regularized factor. One could also attempt to control the scale of the unregularized factor within the framework of Problem 9 by choosing 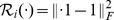 or 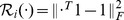 [13]. However, this again requires balancing of 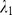 against 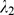 which is difficult, and, moreover, the scale can only be controlled approximately. Another ad hoc approach could be to compensate for the pull of the regularization term by standardizing the column norms of 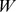 or 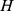 between iterations. This is equivalent to inserting a diagonal matrix 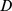 and its inverse between the factors. This operation is safe in conventional NMF because the value of the objective function will not change. With a regularization term, however, column standardization is unsafe: although the value of the fitting term 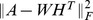 will not change, the value of the regularization term may, meaning the objective function may increase between iterations. To control the scale exactly, [20] proposed a truncated gradient descent method and [21] a multiplicative update method, and studied regularization with respect to sparsity. These methods represent the closest predecessors of our approach and were therefore used as control methods.

When it comes to the convergence, the strongest proved result for conventional NMF is guaranteed convergence to critical points. Some conventional NMF methods always find critical points, for example alternating non-negative least squares. By contrast, regularized NMF methods are less well characterized. To our knowledge, the only regularized NMF method that is known to guarantee critical point solutions is an alternating non-negative least squares method that solves Problem 9 when 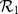 is the squared 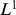 norm and 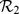 is the 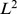-norm [32]. Methods based on Lee-Seung’s multiplicative descent method do not guarantee critical points [13], nor do current exact-scale methods [20], [21].

In conclusion, we have presented a new framework for regularized NMF. Our approach has advantages in that it accommodates for a wide range of regularization terms, guarantees solutions that satisfy necessary conditions for optimality, allows the scale of the solution to be controlled exactly, is computationally efficient, and enables decomposition of gene expression data subject to knowledge priors. Hopefully, this study, along with other efforts, will further the development of methods to analyze complex high-dimensional data.

## Materials and Methods

Microarray data sets generated on Affymetrix microarrays were retrieved from NCBI Gene Expression Omnibus (http://www.ncbi.nlm.nih.gov/gds; accession numbers GSE1159, GSE6891, GSE12417, GSE13159, GSE19784, and GSE28497). Because NMF assumes an additive model, non-log transformed gene expression values were used throughout the experiments. Sets of cell type-specific markers were inferred by use of the d-map compendium containing gene expression profiles of all major classes of blood cells sorted by flow cytometry (Affymetrix U133A arrays) [24]. One set per cell type was inferred by comparing d-map profiles belonging to this cell type to all others using Smyth’s moderated t-test [35], selecting the top 100 probes as markers (results agreeing with those shown were obtained using the top 50, 150, 200 and 250 probes). Gene set enrichment testing was performed using the program RenderCat [25].

### URLs

A C++ implementation is available at http://www.broadinstitute.org/~bnilsson/rNMF.rar.

### Algorithm 1

Pseudocode to optimize a column 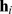 in 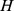, given 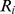, 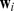, the current 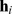, and the proximal point parameter 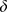. Note that in the if clause, the first condition 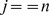 asserts that the program never tries to reach 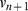 whereas the second asserts that 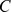 is the minimal value of the partial derivatives. Because 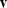 is sorted in descending order, and 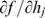 equals 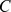 if 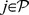 and 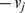 otherwise, it is sufficent to compare 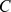 with 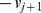.


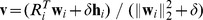



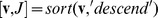



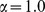


**for** 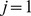 **to** 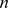 **do**


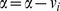



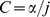


**if** 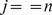 **or** 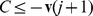 **then**


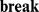


**end if**

**end for**


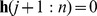



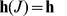


**return** 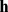


### Algorithm 2

Pseudocode for the complete rNMF procedure with 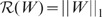. To change the type of regularization, change the 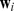 update. Note that the rank-one residual 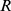 is updated cumulatively to save computations.


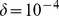



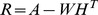


**Repeat**

**for** 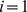 **to** 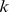 **do**


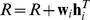



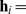
 Algorithm1(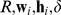)


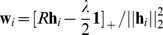



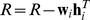


**end for**

**until** stopping criterion is reached

**return** 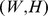

